# Comparative Analysis of Histomorphological Quality and Quantitative Cell Assessment in Formalin-Fixed Paraffin-Embedded and Fresh Frozen Porcine Skin Biopsies

**DOI:** 10.3390/biomedicines14020390

**Published:** 2026-02-08

**Authors:** Lina Winter, Volker H. Schmitt, Friedrich Barsch, Dominic Schwarz, Cristina L. Cotarelo, Christoph Brochhausen

**Affiliations:** 1 Institute of Pathology, Medical Faculty Mannheim, Heidelberg University, Theodor-Kutzer Ufer 1-3, 68167 Mannheim, Germany; 2 Cardiology I, Centre for Cardiology, University Medical Centre, Johannes Gutenberg University of Mainz, Langenbeckstraße 1, 55131 Mainz, Germany; 3 Institute for Exercise and Occupational Medicine, University Hospital of Freiburg, Hugstetter Str. 55, 79106 Freiburg im Breisgau, Germany

**Keywords:** histopathology, methodology, preservation, formalin-fixed paraffin-embedded, paraffin, cryostat, frozen section, morphological quality

## Abstract

**Background:** Efficient tissue preservation methods are critical for accurate and quantitative microscopical examination in histopathology. Quantitative image analysis and cell counts are essential to translational research with direct implications for therapeutic decision-making. This study aims to compare the histomorphological quality of formalin-fixed paraffin-embedded (FFPE) and fresh frozen sections (FFS) in terms of tissue recognizability, physical integrity, as well as cell counts of lymphocytes, granulocytes, giant cells, and blood vessels **Methods:** A total of 142 skin biopsies were analyzed, with 88 FFPE and 54 with FFS. The biopsies were stained with HE and ASD. The sections were evaluated for recognizability and physical appearance, and categorized as either clearly recognizable or indistinctly recognizable, and fully intact, folded, or torn. Suited high-power fields were identified to compare the number of different cell types between the two preservation techniques. **Results:** FFPE showed significantly higher morphological quality than FFS in maintaining both recognizability (88.64% vs. 44.44%, *p* < 0.001) and physical integrity, with more sections remaining fully intact (77.27% vs. 22.22%, *p* < 0.001). Additionally, paraffin sections showed higher counts of lymphocytes and blood vessels (both *p* < 0.001) with significant statistical differences. **Conclusion:** The findings suggest that FFPE provides superior tissue preservation compared to FFS, particularly in maintaining structural integrity and cellular detail. This study underlines the importance of choosing appropriate embedding techniques to optimize histological evaluations, especially in clinical settings where quantitative analyses are crucial.

## 1. Introduction

In the mid-19th century, scientists faced significant challenges in preparing animal soft tissues for microscopic examination due to the lack of suitable embedding techniques [1]. Over the years, several approaches were evaluated, including the hardening of the tissue with alcohol or fixatives, or freezing them outdoors during winter [1,2]. Additionally, substances like beeswax were considered as potential embedding materials [1]. Although Edwin Klebs is often acknowledged as the inventor of paraffin embedding in 1867, he diverged from this technique in 1869 due to the material’s insufficient infiltration into tissues. However, Wilhelm His Senior took up the method and refined it by the integration of dehydration and clarification steps [1]. Nowadays, more than 150 years later, paraffin embedding remains the histological standard in diagnostic histopathology. Interestingly, the basic steps are remarkably similar to the technique of His Senior [1]. Formalin-fixed paraffin-embedded (FFPE) tissue allows for the creation of approximately 3 to 8 μm thin sections, which can be stored at room temperature [3]. However, paraffin embedding may lead to a loss of the tissue’s water content resulting in shrinkage, and to a loss of adipose tissue components due to the use of lipid-soluble solvents [4]. In contrast, the cryostat preserving method involves immersing fresh organ pieces in liquid nitrogen to solidify them. Subsequently, the sections are prepared using an appropriate cryo-microtome. Due to technical reasons, these sections are slightly thicker than those from paraffin embedding, typically ranging from 5 to 10 μm, and shrinkage also appears [5,6]. In a previous histopathological analysis, we clearly demonstrated that the bronchial wall thickness was significantly smaller in frozen sections compared to paraffin sections indicating that paraffin sections exhibited less shrinkage [7]. The advantage of cryopreservation includes preservation of the lipid components and its time efficiency allowing for an intraoperative histological diagnosis within 5 to 10 min [8]. Thus, it is a less time-consuming process than paraffin embedding which lasts approximately 4 to 24 h.

Frozen sections prepared from cryopreserved tissue are an integral part of the routine diagnostic. They enable rapid assessment of tissue samples and guide intraoperative decision-making, for example for margin evaluation. Due to their time efficiency and broad availability, cryostat sections are widely accepted in daily clinical practice [8]. At the same time, it has been demonstrated that the diagnostic reliability of frozen sections is not uniform and depends on tissue type, lesion characteristics, and morphological architecture [9,10]. In particular, diagnostic challenges related to freezing artifacts, altered tissue architecture, and limited cellular definition have been described, resulting in entity-dependent diagnostic uncertainty [9,10]. Importantly, the existing literature has primarily focused on qualitative diagnostic endpoints, such as concordance between frozen section and final histology and sensitivity and specificity for malignancy detection and margin assessment [10,11,12,13].

These diagnostic limitations are especially present in tissue characterized by diffuse growth patterns, poorly defined cell borders, or abundant extracellular matrix components. Tumor entities with dispersed single-cell growth, such as poorly cohesive or signet-ring cell carcinomas, exemplify situations in which cells may be present in frozen sections but remain difficult to distinguish from surrounding stromal or inflammatory elements due to their morphology and the effects of freezing-related artifacts [14]. Such conditions illustrate that limited morphologic recognizability, besides tissue sampling, can be a critical limiting factor in frozen section evaluation.

While these challenges are well described in the context of qualitative intraoperative diagnosis, their implications for quantitative histopathological analyses have received less attention. Reliable tissue preservation is fundamental for high-quality histopathological evaluation. In modern translational research, quantitative image analysis and cell-based measurements are increasingly used to characterize inflammatory responses, angiogenesis, and tissue remodeling, with direct implications for therapeutic decision-making [15,16]. While FFPE and cryostat sections are both widely used in routine diagnostics and research, there is limited systematic evidence directly comparing their histomorphological quality and suitability for quantitative image analysis under standardized conditions. In particular, it remains unclear to what extent differences in tissue preservation, processing, and section quality may influence quantitative cell counts and structural assessments.

This study aims to systematically compare FFPE and frozen sections regarding the histomorphologic quality and the applicability to quantitative image-based cell assessments within skin biopsies treated with wound dressings.

## 2. Materials and Methods

### 2.1. Porcine Model

The present study is part of an animal study that investigated wound healing after the transplantation of foreign skin material and is extensively described in previous research [17]. Because frozen sections are rarely performed for purely inflammatory processes in routine pathology, an animal model was deemed necessary. Porcine skin closely resembles human skin in terms of thickness, structure, and wound healing characteristics, making it a well-established and validated model for dermatological and wound healing research [18]. This model enabled paired FFPE and FFS sampling from identical anatomical sites under standardized inflammatory conditions, allowing a comparison of both preservation methods. Specifically, the model was applied to compare the histomorphologic quality and the cell count of different cell types FFPE and FFS.

The study was approved under the reference number V3-2347-A-6-19-2013 in accordance with the German provisions of the Animal Welfare Act and the European guideline Directive 90/385/EEC. Two pigs were included in the present experiment. Each pig was marked with 20 square fields, from which full-thickness skin excisions were performed. These excised parts were then treated with dermal replacement materials. The harvested split-thickness skin was meshed and subsequently transplanted onto the dermal replacement materials. Five days after the implantation of the matrices, two biopsies were taken from each marked square field and subsequently processed using cryostat or paraffin embedding methods for a direct comparative analysis. One of the biopsies was prepared for cryostat sectioning (5 μm), while the other one was embedded in paraffin after fixation in 4% buffered formalin. Subsequently, paraffin sections of 5 μm thickness were performed. Frozen and paraffin-embedded sections were stained with hematoxylin and eosin (HE) [19] and naphthol AS-D chloroacetate esterase (ASD) according to standardized protocols [20]. The ASD staining was used to support the histochemical identification of granulocytes. Only technically suitable samples were included in the final analysis.

### 2.2. Histopathological Evaluation

To assess the morphological quality of the biopsies, paraffin and frozen sections were categorized according to their overall histomorphological recognizability, with a particular emphasis on cellular delineation into “clearly distinguishable” and “indistinctly recognizable”. Biopsies rated as “clearly distinguishable” exhibited well-defined structures with clearly distinct cell nuclei and cell membranes, enabling precise assessments of cell integrity and straightforward assessment of the specific cell types. In contrast, sections of the category “indistinctly recognizable” were characterized by poor definition of cell membranes and less structural details with view to cell nucleus morphology. Furthermore, each biopsy’s physical appearance was also evaluated regarding its tissue integrity, including “fully intact”, “folded”, or “torn” biopsies.

In the next step, high-power fields were identified to assess the number of specific cell types, including blood vessels, lymphocytes, granulocytes, and giant cells. High-power fields, defined as the microscopic field of view at 400× total magnification, were selected in regions with well-preserved tissue architecture and representative inflammatory infiltrates. Whenever possible, anatomically corresponding regions were chosen in paired paraffin and cryostat sections.

The evaluators were not blinded to the preservation method, as the morphological differences between FFPE and FFS are apparent and prevent effective blinding. To minimize potential observer bias, all evaluations were performed independently by two trained experts (D.S. and V.H.S.) using predefined criteria. Representative images of the tissue were taken using a high-resolution scanner (3DHISTECH Ltd. Pannoramic slide scanner 250, Budapest, Hungary).

### 2.3. Statistical Evaluation

IBM SPSS Statistics (Version 30.0.0, IBM Corp., Armonk, NY, USA) was used for statistical evaluation. In terms of tissue recognizability and physical integrity, the Chi-squared test was used to compare paraffin and cryostat sections. A t-test was applied to compare the cell counts of lymphocytes, giant cells, granulocytes and blood vessels between paraffin and cryostat. Differences were considered statistically significant at *p* < 0.05 and highly significant at *p* < 0.001.

## 3. Results

### 3.1. Morphological Quality

A total of 88 FFPE and 54 cryostat sections were analyzed regarding their histomorphological quality, namely recognizability and physical integrity. The results, shown in Table 1, indicate statistically significant differences in both categories between the two methods (*p* < 0.001). Paraffin sections demonstrated a significantly higher percentage of clear recognizability (88.64%) compared to cryostat (44.44%). Correspondingly, paraffin sections were more likely to remain fully intact (77.27%) compared to the cryostat sections (22.22%). In contrast, cryostat sections showed significantly higher percentages of folded (11.11% in cryostat vs. 1.14% in paraffin) or torn morphology (66.67% in cryostat vs. 21.59% in paraffin).

**Figure 1:**
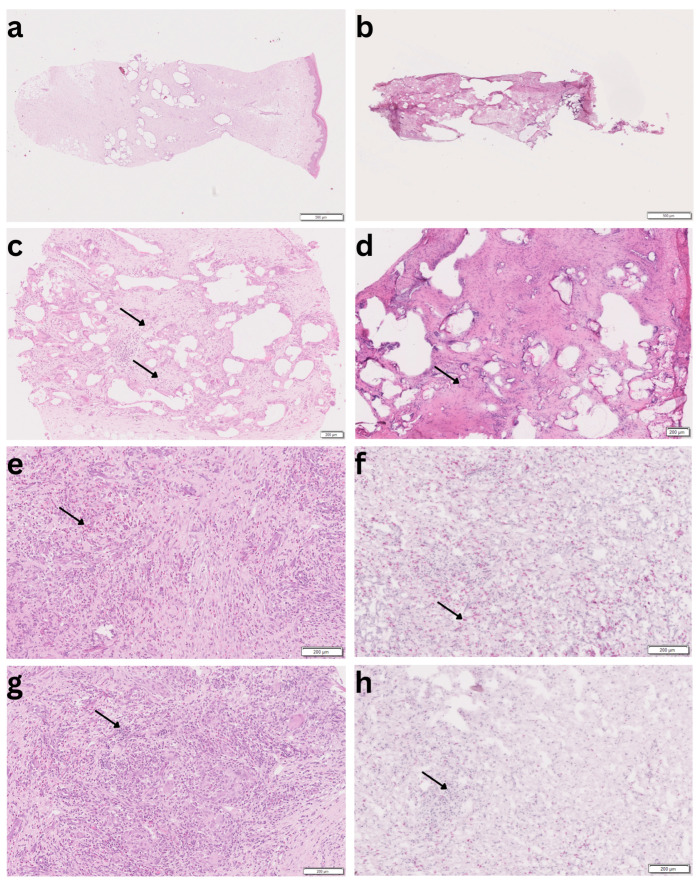
Morphological quality of paraffin sections in the left column and cryostat sections in the right column. (**a**,**b**) Physical appearance of the tissue sections, magnification 4× (HE); (**c**,**d**) blood vessels (HE, magnification 20×, representative arrow); (**e**,**f**) granulocytes (ASD, magnification 40×, representative arrows); (**g**,**h**) lymphocytes (HE, magnification 40×, representative arrows).

Figure 1 shows exemplary images of paraffin and cryostat sections.

### 3.2. Cell Counts

**Figure 2:**
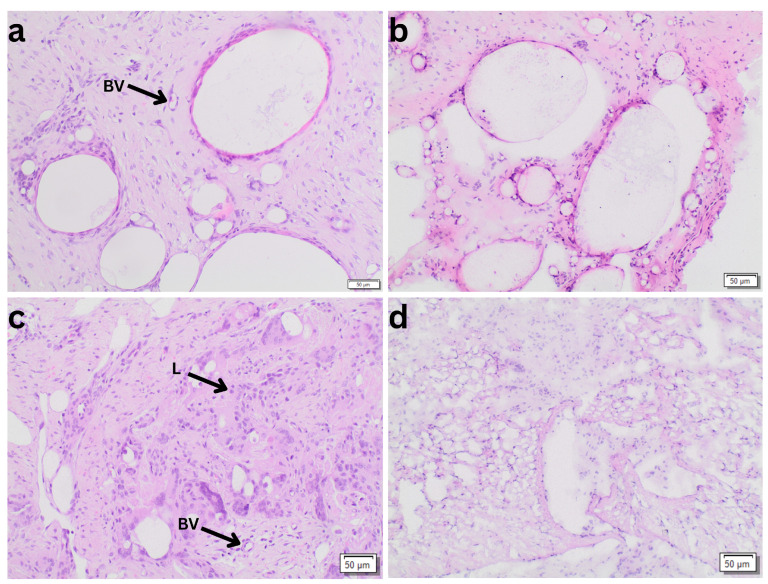
Representative histological comparison of formalin-fixed paraffin-embedded (FFPE) and cryostat sections at 100× magnification. (**a**,**c**) FFPE sections demonstrating well-defined tissue architecture with clearly recognizable small blood vessels (BV) and inflammatory cells, including lymphocytes (L); (**b**,**d**) corresponding cryostat sections showing reduced cellular and structural definition.

In the comparison of tissue preservation methods, in the HE staining, a total of 657 and 496 high-power fields, and in ASD staining, a total of 388 and 401 high-power fields were analyzed in paraffin and cryostat biopsies respectively. Paraffin embedding demonstrated significantly higher mean numbers of lymphocytes (1.6086 ± 1.30447, *p* < 0.001) and blood vessels (1.4635 ± 1.01273, *p* < 0.001) compared to the cryostat technique (lymphocytes: 0.8788 ± 0.63909; blood vessels: 0.7055 ± 0.52993). The 95% confidence intervals (lymphocytes: 0.397 to 1.083; blood vessels: 0.514 to 1.039) confirm the robustness of these findings. In contrast, no significant differences were observed in the counts of giant cells (paraffin: 0.7391 ± 1.19581 vs. cryostat: 0.5347 ± 0.76417, *p* = 0.300) and granulocytes (paraffin: 0.5977 ± 1.68495 vs. cryostat: 0.5111 ± 1.71541, *p* = 0.625). The results are demonstrated in Table 2. Figure 2 illustrates representative FFPE and cryostat sections at 100× magnification. At higher magnification, FFPE sections clearly demonstrate superior morphologic clarity, allowing more reliable identification of small blood vessels as well as individual inflammatory cells, including lymphocytes and granulocytes, compared with corresponding cryostat sections.

## 4. Discussion

As expected, the present analysis clearly demonstrates that FFPE sections display better histomorphological quality compared to FFS. Thus, our results underline the FFPE sections as gold standard in histological analyses. Our results reveal significant differences in lymphocyte and blood vessel counts between the two methods, which is crucial for quantitative image analyses, especially for research purposes. Higher cell counts observed in FFPE sections are most likely due to improved morphological preservation and clearer delineation of individual cells [14]. This facilitates reliable identification and quantitative assessment. In cryostat sections, relevant cell types are frequently present but often display indistinct cell borders and reduced structural clarity. Nevertheless, potential influences related to tissue processing cannot be entirely excluded and should be considered when interpreting quantitative differences between preparation methods. In this context, it is noteworthy that previous histopathological analyses demonstrated less overall tissue shrinkage in FFPE sections compared with frozen sections [7]. This supports the interpretation that differences in quantitative cell counts are primarily driven by morphological recognizability rather than global tissue shrinkage alone.

In pathological routine diagnosis, it is an incontrovertible fact that FFPE is the morphologically superior method compared to cryostat sections. However, there is limited evidence that systematically describes and quantifies this histomorphological benefit of FFPE. The essential application of cryostat sections is the rapid intraoperative diagnosis, especially during oncologic surgeries. In this context, the ability to accurately define tumor margins during surgery is crucial for efficient oncologic resection [21,22]. In several tumor entities, cryostat sections are well-established to assess tumor margins and guide surgical decisions intraoperatively. Despite the common use of intraoperative frozen sections during surgeries for oral cavity squamous cell carcinoma, a recent study has clearly demonstrated their limited sensitivity at only 11% [11]. The researchers attributed this low sensitivity to the complex three-dimensional relationship of tumor margins to the specimen’s periphery and the generally low incidence of positive final tumor margins [11]. In general, frozen sections offer rapid feedback to the surgeon but sometimes lack the detail needed to clearly outline tumor margins. This can result in incomplete tumor excision or the need for further surgical procedures, clearly showing the technical limitations of the method. However, it should be considered that this fact does not represent a result of the cryo technique itself, but of the unique tumor characteristics. In our own study, we compared the different stained morphological components of the tissue sections in both methods.

Intraoperative lymph node assessment showed a specificity of 100%, thus a false-positive rate of almost 0 [23]. However, studies reported a sensitivity of 57 to 74%, indicating that some tumor-positive lymph nodes are not detected during rapid diagnostics, but can be identified in subsequent FFPE or IHC analyses. The authors attributed this fact to the high variability of specific protocols [23]. Specifically, Cserni et al. reported that there exist 123 different protocols for 240 European units that process sentinel lymph node biopsies [24]. In this context, high quality of the protocol with a high extent of lymph node examination results in a higher concordance of intraoperative frozen sections and subsequent histological examinations. Furthermore, Layfield et al. stated that the performance of frozen sections leads to irreversible tissue loss. In particular, lobular carcinoma is highly challenging to detect in frozen sections, since tumor cells are often cytologically bland and have an infiltrative growth characteristic. Thus, cryostat sections represent a compromise that needs careful consideration, offering significant time efficiency but also presenting a risk of underdiagnosing [23]. In this context, Cotarelo et al. demonstrated that a dual approach of touch imprint cytology and subsequent frozen section significantly enhance the detection of sentinel lymph node macrometastases [25].

Another relevant aspect is the application of deep learning methods to frozen sections. On the one hand, artifacts resulting from the freezing process make automated evaluation by AI models more difficult. In addition, many models are primarily trained on FFPE tissue, as there are significantly fewer publicly available data sets for FFS in comparison. This limits the training and the generalizability of corresponding models. However, Gorman et al. showed that models trained on FFS data can be more robust against artifacts despite the initially lower image quality, and can be better transferred to other preparation types than models based solely on FFPE [26].

Regardless of these challenges regarding cryo-section, there are specialties in which performing cryo-sections is considered as the method of choice, which suggests that the selection of the method should be context-adapted. In microbiota research, for example, frozen sections are preferred, as the quality of the DNA is better preserved [27]. Cryostat methods are also frequently used in materials science, as it is considered more protective regarding the preservation of biografts, for example, in terms of thermosensitivity. However, we clearly demonstrate in our present study that FFPE can also be used for wound dressings and leads to histomorphologically superior results. A particular focus in the application of biomaterial in situ is examining tissue reactions. This includes studying inflammatory responses or the integration of materials via angiogenesis [17,28]. In this context, it is highly relevant that we were able to show that significantly fewer blood vessels and lymphocytes are found in cryosections than in FFPE. This could be due to the fact that the cell boundaries of lymphocytes are not clearly recognizable in the frozen sections and grouped lymphocytes are thus more difficult to separate. Therefore, the evaluation of cryosections could lead to an underestimation of the extent of inflammation and angiogenesis. Interestingly, we could not show a significant granulocyte count variation between FFPE and cryostat.

The preparation of frozen sections does not require formalin fixation, which can impair the natural conformation of the cellular structures and reduce the antigen accessibility. However, cryostat sections are susceptible to the formation of artifacts due to the rapid freezing in liquid nitrogen. The snap-freezing and the evaporation of liquid nitrogen when it comes into contact with the warm tissue leads to the formation of large ice crystals that can damage cellular structures. Therefore, isopentane is often used as a freezing medium. In this context, Ghag et al. have developed a protocol that prevents freeze-fractures in small muscle fibers by using frozen isobutane instead of liquid isobutane. This example illustrates that the protocol has a relevant impact on histomorphological quality. Correspondingly, Ryu et al. have established the so-called “sticker method” to enhance the histomorphology of cryostat sections. The tissue samples are embedded in optimal cutting temperature (OCT) compound and rapidly frozen with an attached adhesive film to preserve morphology. Following that, the samples are transferred to microscope slides where the adhesive film can be removed, exposing the well-preserved frozen tissue for detailed histological examination [29]. For our present project, we used our standard frozen section protocol with snap-freezing of the fresh tissue. Optimizing our protocol might potentially lead to an improvement in the morphological quality of the frozen sections. Therefore, further evaluations and refinements are warranted.

However, there remains considerable debate within the literature regarding the preservation of molecular markers for histochemical analyses. In this context, Hira et al. found that FFPE sections show superior tissue morphology, but cryostat sections exhibit better antigen preservation in immunohistochemistry [30]. Correspondingly, Ananian et al. highlighted better preservation and more reliable evaluation of DNA structures in cryo-embedded forensic samples compared to those in paraffin [31]. Furthermore, Raymond et al. observed a 10% higher sensitivity in detecting carcinomas with paraffin embedding despite the higher staining intensity of the cryostat method [32]. Conversely, Shimada et al. found no significant differences in the detection of breast carcinoma between the two methods [33]. Finally, Ward et al. identified a reduction in counted nuclei by up to 10% in cryostat compared to paraffin in optical dissector comparisons [34].

Future studies might assess differences in immunohistochemical staining or in RNA and DNA quality, for example, with RIN^e^ and DIN-values as performed in previous research [35].

Kleb’s and His Senior’s pioneering work continues to influence the working routines of pathologists, researchers, and laboratory staff to this day. Nevertheless, innovative technologies are constantly shaping the field of pathology [36,37].

This study has several limitations. First, an unequal number of FFPE and FFS samples (88 vs. 54) was included in the comparative analysis. This imbalance resulted from the inclusion of biopsies of sufficient technical quality for quantitative evaluation, which led to a higher exclusion rate among the cryostat sections. In fact, this circumstance highlights the technical challenges associated with the preparation of cryostat sections. Second, in histological studies like the present, operator skills might affect the quality of tissue sections and potentially lead to operator bias. However, in the present study, all sections were prepared by an experienced laboratory worker trained in paraffin and cryostat methods. This controlled setup helps to ensure that any differences observed between the methods are due to their inherent properties and not due to variations in technique. Therefore, we are convinced that our results accurately reflect the comparative performance of these techniques under consistent conditions.

## 5. Conclusions

In conclusion, our comparative analysis of paraffin-embedded and cryostat-prepared skin biopsies demonstrated that cryostat sections are less suitable for quantitative histological evaluations. While relevant cell types are present in cryostat sections, their morphological recognizability is reduced compared with FFPE sections. This limits reliable cell identification and reproducible quantification. In contrast, paraffin embedding provides superior tissue preservation and cellular definition, thereby facilitating robust quantitative image analysis. For future studies relying on quantitative cell-based assessments, FFPE preparation should therefore be preferred.

## Figures and Tables

**Table 1:** Comparison of morphological quality between paraffin and cryostat sections.

		Paraffin		Cryostat		
		n	%	n	%	Chi-Square *p*-Value
	Total biopsies	88	100	54	100	
Recognizability	Yes (clearly recognizable)	78	88.64	24	44.44	<0.001
	No (indistinctly recognizable)	10	11.36	30	55.56	
Physical appearance	Fully intact	68	77.27	12	22.22	<0.001
	Folded	1	1.14	6	11.11	
	Torn	19	21.59	36	66.67	

**Table 2:** Comparison of different cell types per high-power fields in paraffin and cryostat biopsies.

		Mean	Standard Deviation	Significance (*p*-Value)	95% Confidence Interval
Lymphocytes	Paraffin	1.6086	1.30447	<0.001	0.397, 1.083
	Cryostat	0.8788	0.63909		
Blood vessels	Paraffin	1.4635	1.01273	<0.001	0.514, 1.039
	Cryostat	0.7055	0.52993		
Giant cells	Paraffin	0.7391	1.19581	0.300	−0.144, 0.462
	Cryostat	0.5347	0.76417		
Granulocytes	Paraffin	0.5977	1.68495	0.625	−0.834, 0.504
	Cryostat	0.5111	1.71541		

## Data Availability

The data presented in this study are available on reasonable request from the corresponding author.

## Abbreviations

The following abbreviations are used in this manuscript:

ASD	Naphthol AS-D chloroacetate esterase
DIN	DNA Integrity Number
DNA	Deoxyribonucleic acid
FFPE	Formalin-fixed paraffin-embedded
FFS	Fresh frozen sections
HE	Hematoxylin and eosin
IHC	Immunohistochemistry
OCT	Optimal Cutting Temperature
RNA	Ribonucleic acid
RIN^e^	RNA Integrity Number equivalent
SD	Standard deviation
SPSS	Statistical Package for the Social Sciences
